# Limited TCF7L2 Expression in MS Lesions

**DOI:** 10.1371/journal.pone.0072822

**Published:** 2013-08-20

**Authors:** Alexander Lürbke, Karin Hagemeier, Qiao-Ling Cui, Imke Metz, Wolfgang Brück, Jack Antel, Tanja Kuhlmann

**Affiliations:** 1 Institute of Neuropathology, University Hospital Münster, Münster, Germany; 2 Neuroimmunology Unit, Montreal Neurological Institute, McGill University, Montreal, Canada; 3 Department of Neuropathology, University Medical Center, Göttingen, Germany; Université Pierre et Marie Curie-Paris6, INSERM, CNRS, France

## Abstract

Multiple sclerosis is the most frequent demyelinating disease in the human CNS characterized by inflammation, demyelination, relative axonal loss and gliosis. Remyelination occurs, but is frequently absent or restricted to a small remyelinated rim at the lesion border. Impaired differentiation of oligodendroglial precursor cells is one factor contributing to limited remyelination, especially in chronic MS. TCF7L2 is an oligodendroglial transcription factor regulating myelin gene expression during developmental myelination as well as remyelination. TCF7L2 binds to co-effectors such as β-catenin or histone deacetylases and thereby activates or inhibits the transcription of downstream genes involved in oligodendroglial differentiation. To determine whether TCF7L2 can be used as a marker for differentiating or myelinating oligodendrocytes, we analyzed the expression patterns of TCF7L2 during myelination and remyelination in human and murine CNS tissue samples. Here, we demonstrate that marked expression of TCF7L2 in oligodendrocytes is restricted to a well defined time period during developmental myelination in human and mouse CNS tissue samples. In demyelinating diseases, such as multiple sclerosis, TCF7L2 is reexpressed in oligodendrocytes in a subset of MS patients, but is also present in tissue samples from patients with non-demyelinating, inflammatory diseases. Furthermore, TCF7L2 expression was also detected in astrocytes. HDAC2, a potential binding partner of TCF7L2 that promotes oligodendroglial differentiation and myelination, is expressed in the majority of oligodendrocytes in controls and MS tissue samples. In summary, our data demonstrate that the expression of TCF7L2 in oligodendrocytes is limited to a certain differentiation stage; however the expression of TCF7L2 is neither restricted to the oligodendroglial lineage nor to (re-)myelinating conditions.

## Introduction

In Multiple sclerosis (MS) loss of axons is a prominent feature that correlates with neurological impairment [1–4]. In vivo imaging findings support the importance of axonal loss for persistent disability [5,6]. Demyelination contributes to axonal damage by increased vulnerability to immune mediated injury and lack of trophic support from myelin sheaths [7,8]. Furthermore, lack of remyelination is associated with increased axonal damage and loss in demyelinating animal models and MS lesions [9–11]. Promotion of remyelination appears to be an attractive neuroprotective strategy in demyelinating diseases. However, although remyelination is a frequent event in early MS lesions, it is limited in chronic lesion stages [12,13]. Only about 20% of chronic MS lesions show extensive remyelination of more than 50% of the lesion area [14]. Limited remyelination despite the presence of OPCs in the majority of chronic MS lesions suggests that a differentiation block of oligodendrocytes contributes to remyelination failure [15–17].

Differentiation and myelination of oligodendrocytes is regulated by a broad range of transcriptional (e.g. Olig1, Olig2, Nkx2.2, Sox10) and postranscriptional factors (e.g. microRNAs, histone modifications). TCF7L2 is a transcriptional factor that has been found to be transiently expressed in oligodendrocytes during myelination [18]. Mice lacking TCF7L2 have OPCs but fail to generate mature oligodendrocytes. TCF7L2 is an effector of the wnt/β-catenin-pathway and the expression of members of the wnt/β-catenin-pathway and TCF7L2 has been observed in MS lesions. Enforced constitutive expression of β-catenin inhibited myelination and differentiation of oligodendrocytes leading to the suggestion that a disturbed wnt/β-catenin-pathway may contribute to remyelination failure in chronic MS lesions [18,19]. Alternatively, TCF7L2 may bind to histone deacetylases and promotes differentiation of oligodendrocytes [19].

Here we demonstrate that during myelination in the human and mouse CNS TCF7L2 expression is limited to a defined differentiation stage of oligodendroglial lineage cells. In early MS lesions TCF7L2 expression is a relatively rare phenomenon and most prominent in periplaque white matter and remyelinating lesion areas whereas in chronic MS lesions TCF7L2 expression was completely absent. However, reexpression of TCF7L2 is neither limited to the oligodendroglial lineage nor to demyelinating lesions since we found a marked expression of this transcription factor in oligodendrocytes and astrocytes in inflammatory, non demyelinating diseases.

## Materials and Methods

### 1. Tissue samples

#### Mouse tissue samples

For developmental studies P10, P21, 8 weeks and 8 months old mice (n = 3 to 5 each) were sacrificed.

For the cuprizone experiments six- to eight-week-old male C57Bl/6 mice were purchased from Charles River (Sulzfeld, Germany). Animal handling and experiments were conducted according to the German animal protection laws and approved by the responsible governmental authority (Bezirksregierung Braunschweig; AZ G86.06 and LANUV Nordrhein-Westfalen (AZ 8.87-50.10.37.09.221)).

Cuprizone was prepared by adding 0.25% (w/w) cuprizone (Sigma) to powdered chow. Seven to eight week old C57Bl/6 mice were fed with cuprizone for up to 42 days. Mice were sacrificed 21, 42, 52 and 70 days (n = 3 each) after onset of cuprizone diet. Control animals (n = 3) received powdered chow without cuprizone. Mice were sacrificed under deep anaesthesia and perfused intracardially with ice-cold phosphate-buffered saline (PBS) followed by 4% paraformaldehyde (PFA) perfusion. Brains (cuprizone experiments and developmental studies) and spinal cords (developmental studies) were removed and embedded in paraffin.

Four µm thick sections were stained with haematoxylin and eosin (HE) and Luxol-fast blue (LFB).

#### Human tissue samples

We retrospectively investigated 53 brain tissue samples from 45 MS patients (33 biopsies/ 37 tissue blocks, 12 autopsies/ 18 tissue blocks), 8 tissue samples from 8 patients with other inflammatory non demyelinating diseases (all biopsies), 17 controls without neurological diseases (all biopsies) and 22 developmental tissue samples (30^th^ gestation week to 48 months) (all autopsies). None of the study authors was involved in decision-making with respect to biopsy or autopsy. Patients’ details (sex, age, disease duration) are summarized in Table 1. Paraffin embedded MS autopsy cases were derived from a collection of multiple sclerosis autopsies collected at the Montreal Neurological Institute, McGill University, Montreal, Canada. The study was approved by the Ethics Committee of the University of Göttingen and Münster.

**Table 1:** Patient details.

Patient	sex	Age	neuropathological diagnosis	Disease duration prior to biopsy or autopsy/ disease course at follow up
1	F	38 years	inflammatory demyelinating lesion, consistent with MS	nk/nk
2	M	44 years	inflammatory demyelinating lesion, consistent with MS	nk/nk
3	F	26 years	inflammatory demyelinating lesion, consistent with MS	nk/nk
4	F	52 years	inflammatory demyelinating lesion, consistent with MS	nk/nk
5	M	57 years	inflammatory demyelinating lesion, consistent with MS	nk/nk
6	F	45 years	inflammatory demyelinating lesion, consistent with MS	6 years/nk
7	M	71 years	inflammatory demyelinating lesion, consistent with MS	nk/nk
8	F	60 years	inflammatory demyelinating lesion, consistent with MS	nk/nk
9	M	65 years	inflammatory demyelinating lesion, consistent with MS	19 days/RRMS
10	F	37 years	inflammatory demyelinating lesion, consistent with MS	2.5 months/RRMS
11	M	32 years	inflammatory demyelinating lesion, consistent with MS	58 days/CIS
12	F	20 years	inflammatory demyelinating lesion, consistent with MS	nk/nk
13	F	34 years	inflammatory demyelinating lesion, consistent with MS	nk/nk
14	F	45 years	inflammatory demyelinating lesion, consistent with MS	nk/nk
15	F	28 years	inflammatory demyelinating lesion, consistent with MS	13 days/CIS
16	F	40 years	inflammatory demyelinating lesion, consistent with MS	nk/CIS
17	nk	22 years	inflammatory demyelinating lesion, consistent with MS	9 days/CIS
18	F	37 years	inflammatory demyelinating lesion, consistent with MS	nk/nk
19	M	28 years	inflammatory demyelinating lesion, consistent with MS	nk/nk
20	F	22 years	inflammatory demyelinating lesion, consistent with MS	nk/nk
21	F	31 years	inflammatory demyelinating lesion, consistent with MS	12 days/CIS
22	F	19 years	inflammatory demyelinating lesion, consistent with MS	17 days/CIS
23	nk	nk	inflammatory demyelinating lesion, consistent with MS	9 days/CIS
24	nk	nk	inflammatory demyelinating lesion, consistent with MS	7 days/RRMS
25	F	58 years	inflammatory demyelinating lesion, consistent with MS	nk/nk
26	F	53 years	inflammatory demyelinating lesion, consistent with MS	nk/nk
27	F	17 years	inflammatory demyelinating lesion, consistent with MS	4 days/CIS
28	F	54 years	inflammatory demyelinating lesion, consistent with MS	nk/nk
29	F	42 years	inflammatory demyelinating lesion, consistent with MS	21 days/CIS
30	M	64 years	inflammatory demyelinating lesion, consistent with MS	23 days/CIS
31	F	54 years	inflammatory demyelinating lesion, consistent with MS	nk/nk
32	F	47 years	inflammatory demyelinating lesion, consistent with MS	36 days/CIS
33	M	31years	inflammatory demyelinating lesion, consistent with MS	6 days/CIS
34	M	63 years	MS	18 years/SPMS
35	M	69 years	MS	7 years/SPMS
36	F	74 years	MS	nk/nk
37	M	49 years	MS	nk/nk
38	F	35 years	MS	nk/nk
39	F	70 years	MS	7 years/PPMS
40	F	60 years	MS	14 years/PPMS
41	F	76 years	MS	20 years/nk
42	M	61 years	MS	32 years/SPMS
43	M	66 years	MS	34 years/SPMS
44	M	57 years	MS	29 years/SPMS
45	nk	Nk	MS	nk/nk
46	F	58 years	MS	22 years/ probably PPMS
47	M	76 years	encephalitis	na/na
48	F	52 years	granulomatous encephalitis	na/na
49	F	49 years	*Toxoplasma* encephalitis	na/na
50	F	41years	encephalitis/vasculitis	na/na
51	M	64 years	chronic CNS inflammation	na/na
52	F	77 years	ABRA	na/na
53	M	73 years	encephalitis	na/na
54	M	61 years	encephalitis	na/na
55	M	39 years	control	na/na
56	M	29 years	control	na/na
57	M	Nk	control	na/na
58	M	32 years	control	na/na
59	M	30 years	control	na/na
60	M	40 years	control	na/na
61	F	39 years	control	na/na
62	M	25 years	control	na/na
63	M	64 years	control	na/na
64	M	72 years	control	na/na
65	M	62 years	control	na/na
66	F	61 years	control	na/na
67	F	24 years	control	na/na
68	M	50 years	control	na/na
69	M	63 years	control	na/na
70	M	72 years	control	na/na
71	F	47 years	control	na/na

nk = not known; na = not applicable; CIS clinical isolated syndrome; RRMS = relapsing remitting MS; SPMS = secondary progressive MS; PPMS = primary progressive MS; ABRA = amyloid-beta related angiitis.

### 2. Classification of multiple sclerosis lesions

All lesions fulfilled the generally accepted criteria for the diagnosis of multiple sclerosis [20–22]. Tissue samples from patients with early MS lesions (biopsies) showed the characteristics of active lesions; they were homogenously infiltrated by numerous macrophages. Early MS lesions were derived in general from patients with a short disease duration of days or few months (4 days to 2.5 months); however in one patient the disease duration was six years. In chronic MS tissue samples (autopsies) inflammation was limited to the lesion border (chronic active lesions) or was absent (chronic inactive lesions); these tissue samples were derived from patients with a long disease duration (between 7 and 34 years). In addition, we classified the de- and remyelinating activity as described in detail earlier [23]. Actively demyelinating lesion areas (AD) (biopsies: n = 17, autopsies: n = 0) were located at the plaque border, these areas were partially demyelinated and infiltrated by numerous macrophages containing myelin degradation products, such as MBP or CNP within their cytoplasm. Demyelinated lesion areas (DM) were infiltrated by macrophages and T cells, but macrophages did not contain myelin degradation products (biopsies: n = 7, autopsies: n = 11). In remyelinating/remyelinated areas (RM) (biopsies: n = 15, autopsies: n = 6), thin, irregularly formed myelin sheaths were seen. Remyelinating lesions early during disease course were infiltrated by numerous macrophages and T cells while remyelinating plaques from chronic multiple sclerosis are characterized by a very mild inflammatory reaction. Periplaque white matter (PPWM) (biopsies: n = 25, autopsies: n = 8) showed no signs of demyelination.

### 3. Immunohistochemistry

Tissue specimens were fixed in 4% paraformaldehyde and embedded in paraffin. Tissue samples were cut in 4 µm thick sections that were stained with haematoxylin and eosin and Luxol-fast blue. Immunohistochemical staining was performed with an avidin-biotin technique. After deparaffinization intrinsic peroxidase activity was blocked by incubation with 5% H_2_O_2_ in PBS for 20 min. Non-specific antibody binding was inhibited with 10% FCS in PBS for 25 min. The primary antibodies were rabbit anti-TCF7L2 (1:100) (Cell Signaling, Boston, USA), mouse anti-TCF7L2 (1:300) (Abnova), rabbit anti-HDAC2 (1:200) (Santa Cruz Biotechnology, Santa Cruz, USA), rabbit anti-myelin basic protein (1:1000) (Boehringer Mannheim, Mannheim, Germany), mouse anti-KiM1P (1:5000) (H.-J. Radzun, Department of Pathology, University of Göttingen, Germany), mouse anti-CD3 (Dako) (1:25), rabbit anti-GFAP (1:2000) (Dako, Denmark), mouse anti-GFAP (1:50) (Dako, Denmark), rabbit anti-Olig2 (1:300) (IBL, Spring Lake Park, Minnesota), rabbit anti-Nogo-A (1:750) (Chemicon International, Temecula, CA) and mouse anti-Nogo-A (1:15.000) (11c7, a generous gift from M.E. Schwab, Brain Research Institute, University of Zürich and Department of Biology, Swiss Federal Institute of Technology, Zürich, Switzerland). For double stainings, we combined mouse or rabbit anti-NogoA or anti-Olig2 antibodies with anti-TCF7L2 or HDAC2. A combined avidin-biotin/alkaline phospahtase (AP) technique was used, using biotinylated and AP-conjugated secondary antibodies (1:50) (Dako, Glostrup, Denmark). Alternatively, secondary antibodies conjugated either to Cy2 or Cy3 (1:200) (Jackson ImmunoResearch Laboratories Inc., West Grove, PA) were used.

### 4. Human fetal OPC cultures

For all human tissue samples used in the in vitro studies written informed consent was obtained. Human fetal brain tissue obtained from 15- to 17-gestational week embryos was provided by the Human Fetal Tissue Repository (Albert Einstein College of Medicine, Bronx, NY). Both the Albert Einstein College of Medicine and McGill University institutional review boards approved these studies. A2B5 expressing cells were isolated immuno-magnetically as previously described [24,25]. The purified cells were plated on a matrix of lysed human fetal astrocytes grown on poly-L-lysine-coated plastic coverslips (astrocyte matrix) (Nunc, Rochester, NY) as described previously (8,9). The cultures were grown in Dulbecco’s modified essential medium (DMEM)-F12 supplemented with N1 (Sigma, Oakville, ON), 0.01% bovine serum albumin (BSA), 1% penicillin-streptomycin, B27 supplement (Invitrogen, Burlington, ON), Thyroid hormone (T3, 2 ng/ml, Sigma, Oakville, ON), Platelet-derived growth factor AA (PDGF-AA, 10 ng/ml, Sigma, Oakville, ON) and Basic fibroblast growth factor (bFGF, 20 ng/ml, Sigma, Oakville, ON).

### 5. Immunocytochemistry

For assessment of cell surface markers, cells were stained with monoclonal antibody O4 (1:50, Hybridoma from Dr. Timothy Kennedy) for 30 min at 4°C then fixed with 4% paraformaldehyde for 10 min at 4°C, followed by blocking with HHG (1 mM HEPES, 2% horse serum, 10% goat serum, Hanks’ balanced salt solution) for 10 min. Cultures were incubated with Goat anti-mouse monoclonal IgM-FITC (1:100, Jackson ImmunoResearch, Westgrove, PA) for 30 min at 4°C. Cells were further stained with polyclonal anti-TCF7L2 (1:100, Cell signaling, Boston, USA) antibody for 1 hour followed by polyclonal goat anti rabbit Cy3 secondary antibody (1:250, Jackson ImmunoResearch) 45 min at room temperature. Antibody isotype controls showed low nonspecific staining (data not shown). Cell nuclei were stained with Hoechst 33258 (1:1000, invitrogen) for 10 min at room temperature. Slides were mounted using gel/mount (Biomeda Corporation, Foster City, CA), and stains were visualized using epifluorescent microscopy (Leica, Montreal, QC) and OpenLab imaging software (OpenLab, Florence, Italy).

### 6. Morphometry and statistics

The numbers of TCF7L2, OLIG2 and NOGOA-positive cells stained with the corresponding antibodies were determined in at least 10 standardized microscopic fields of 10.000 µm^2^ each defined by an ocular morphometric grid. In the text and figures, the mean number of cells/mm^2^ ± SEM is given. For statistical analysis, Student *t*-tests were performed; Bonferroni-corrected one-way or two-way ANOVA tests were performed for multiple comparisons. All tests were classified as significant if the *P*-value was < 0.05. The GraphPad PRISM™ software was used (Graph Pad Software, Inc., San Diego, CA, USA) for these analyses.

## Results

### 1. TCF7L2 expression during murine CNS myelination and toxic demyelination

#### CNS myelination

**Figure 1:**
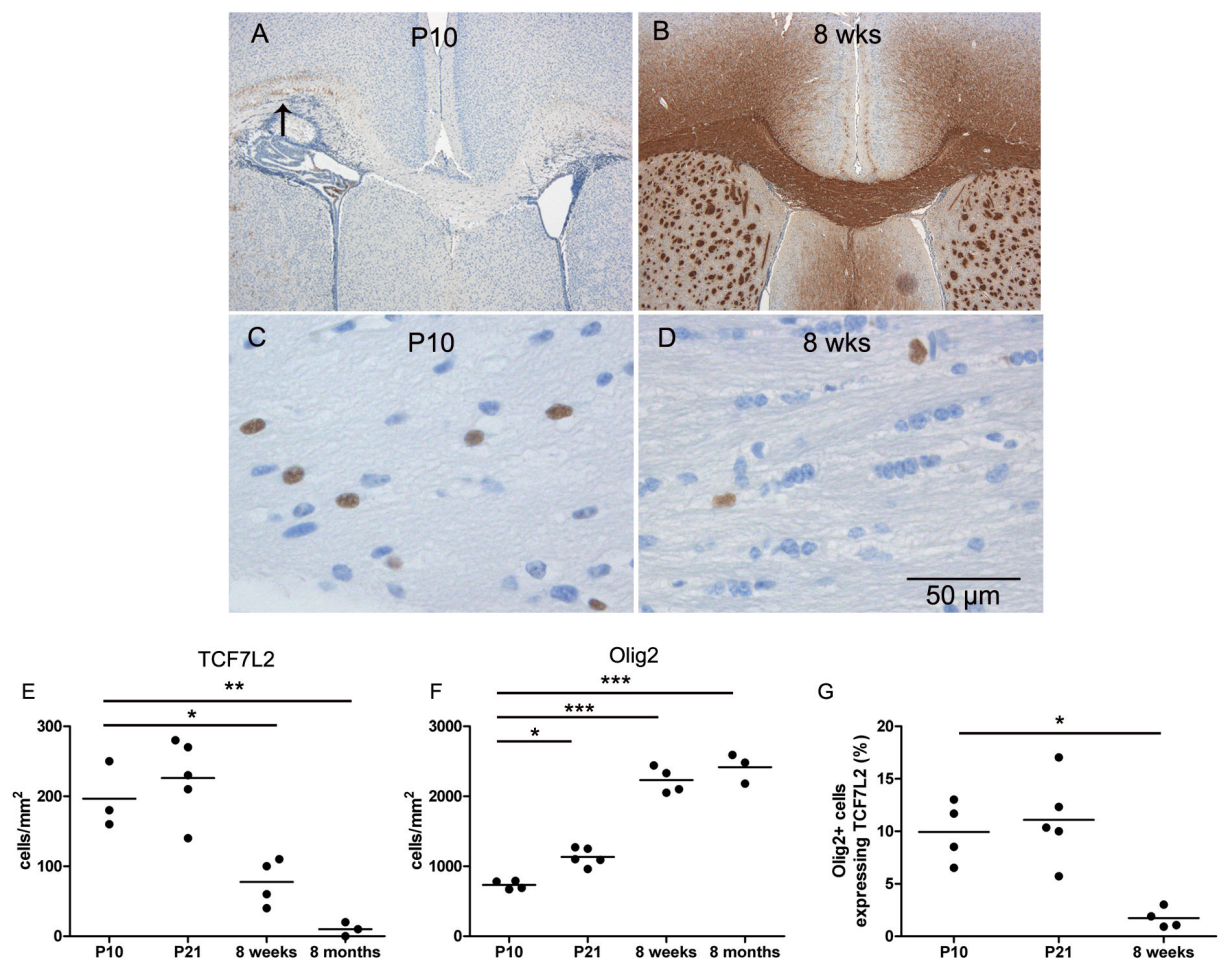
Expression of TCF7L2 during myelination in the CNS of mice. At P10 single myelinated axons are observed in the lateral parts of the corpus callosum (arrow) (**A**) whereas in adult mice myelination is complete (**B**). High numbers of TCF7L2 expressing cells were observed in P10 mice (**C**), but only few TCF7L2-positive cells were found in adult mice (**D**) as quantified in the diagrams (**E** and **F**). The percentage of OLIG2 positive cells expressing TCF7L2 decreased significantly in adult mice (**G**).

Myelination was examined using immunohistochemistry for MBP. At P10 myelination was patchy whereas at P21 myelination was almost complete compared to 8 weeks old adult mice (Figure 1A and B). To determine the expression of TCF7L2 during myelination, we quantified the expression of TCF7L2-positive cells in the corpus callosum of P10, P21, or adult mice (8 weeks and 8 months). We observed a decrease of TCF7L2-positive cells over time with significantly higher numbers at P10 (197 +/- 27 cells/mm^2^) and P21 (226 +/- 25 cells/mm^2^) compared to 8 weeks (78 9 +/- 17 cells/mm^2^) or eight months old mice (10 +/- 6 cells/mm^2^) (Figure 1C–E). The numbers of cells expressing OLIG2, a marker expressed by oligodendroglial lineage cells, increased significantly from P10 to P21 and peaked in adult animals (Figure 1F). Double stainings for TCF7L2 and OLIG2 revealed that almost all TCF7L2-positive cells were OLIG2 positive, only in two out of 13 mice we observed single TCF7L2-positive cell which did not express OLIG2. The number of OLIG2 positive cells expressing also TCF7L2 decreased from approximately 10% at day P10 and P21 to less than 2% at 8 weeks of age (Figure 1G).

#### Toxic demyelination

**Figure 2:**
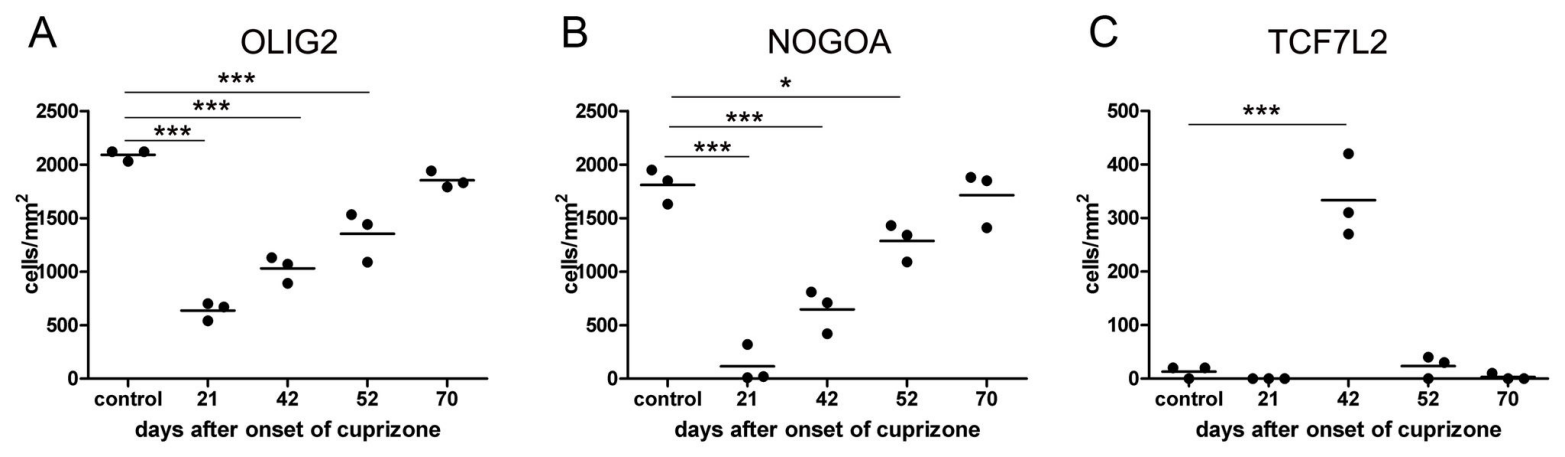
Expression of TCF7L2 during remyelination in the CNS of mice. Mice were fed with 0.25% cuprizone for six weeks and the numbers of oligodendroglial lineage cells were quantified at the indicated time points. Lowest numbers of OLIG2 positive cells were observed 21 days after onset of cuprizone diet; the increased numbers at the end of the cuprizone diet (at 42 days) suggest a recruitment of OPCs during ongoing demyelination (**A**). NOGOA is expressed by mature oligodendrocytes; lowest numbers of NOGOA positive cells were as well found at day 21 (B). High numbers of TCF7L2 expressing cells were only detected at day 42 (C).

Feeding of mice with a cuprizone diet (0.25%) led to oligodendroglial cell death and subsequent demyelination. Oligodendroglial cell death started already after four days of cuprizone diet and reached its peak about three weeks after onset of cuprizone diet [26,27]. To identify oligodendroglial lineage cells we used two different markers. Whereas NOGOA expression is restricted to mature oligodendrocytes OLIG2 is detected in all cells of the oligodendroglial lineage [28]. Although maximal demyelination was observed after six weeks of cuprizone, lowest numbers of NOGOA and OLIG2-positive cells were observed 21 days after onset of cuprizone diet with an increase during the following weeks demonstrating the recruitment of oligodendroglial lineage cells already during the cuprizone diet (Figure 2A and B). Significant upregulation of TCF7L2 was most prominent at day 42 after onset of cuprizone induced demyelination indicating that TCF7L2 is only expressed at a certain stage of oligodendroglial differentiation (Figure 2C).

### 2. TCF7L2 expression during human CNS myelination

**Figure 3:**
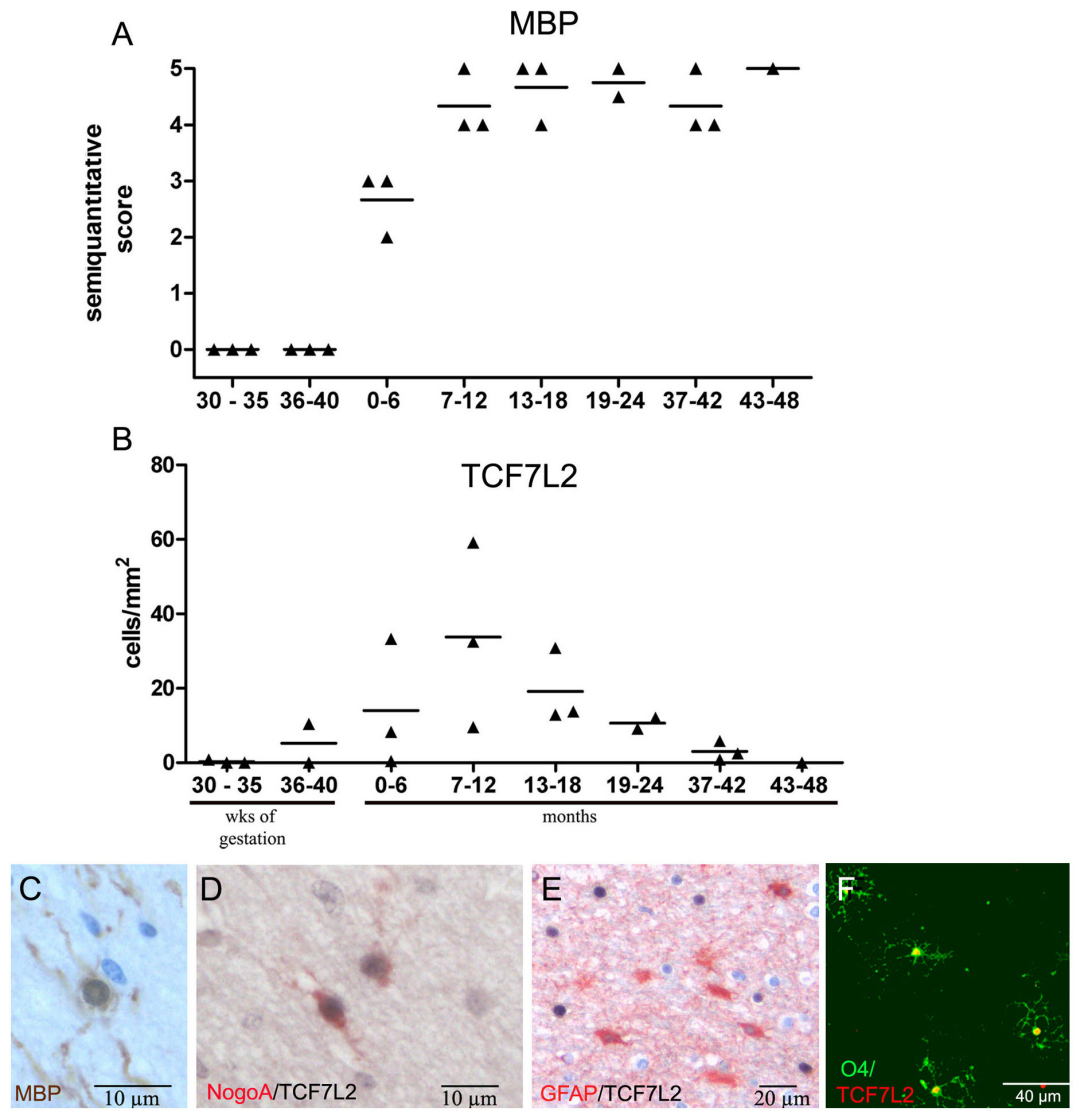
Myelination and expression of TCF7L2 in the human CNS. The extent of myelination in human frontal lobes was quantified using a semiquantitative score. Between 30 and 40 weeks of gestation no MBP-positive axons were found. Myelination became first obvious between 0 and 6 months after birth (**A**). First TCF7L2-positive cells were detected at the end of gestation with maximal numbers between 7 and 12 months after birth. Afterwards, the numbers of TCF7L2-positive cells decreased quickly (**B**). At 6 months after birth numerous myelinating oligodendrocytes were observed (immunohistochemistry for MBP) (**C**). Many TCF7L2 positive cells also expressed NOGOA (double immunohistochemistry for NogoA (red) and TCF7L2 (black) (**D**) but not GFAP (double immunohistochemistry for TCF7L2 (black) and GFAP (red)) (**E**). TCF7L2 was also expressed in human fetal oligodendrocytes in vitro (green O4, red TCF7L2) (**F**).

To determine whether TCF7L2 follows a similar time course during human myelination as in mouse CNS we stained cerebellar and frontal lobe tissue specimens from fetuses (gestation week 32 to 40) and newborns and infants (2 to 48 months) for MBP to determine the course of myelination in the human CNS during development. As expected, myelination in the cerebellum preceded myelination of the frontal lobe (data not shown). Whereas the cerebellum showed already significant myelination at gestation week 32, myelination of the frontal lobes started around birth and was completed approximately 8 months after birth (Figure 3A and C). In frontal lobes, first TCF7L2 expression was observed between the 36^th^ and 40^th^ week of gestation, the highest number of cells expressing TCF7L2 was detected between months 7 and 12 after birth (Figure 3B). Afterwards, TCF7L2 expression decreased and no expression was found 48 months after birth. In white matter tissue specimens from adults (aged between 29 and 64) only single TCF7L2 positive cells were found (mean 5 +/-2 cells/mm^2^; range 0-17/mm^2^, n =9). Double stainings with NOGOA demonstrated that between 50 and 100% (mean 78 +/-6%) of TCF7L2-positive cells also expressed NOGOA in developmental tissue sampes (3D). Similarly, primary OPCs cultured from fetal human CNS expressed TCF7L2 as well (Figure 3F). To determine whether astrocytes express TCF7L2 we performed the appropriate double stainings. However, no TCF7L2-positive astrocytes were detected in developmental tissue samples (Figure 3E). Together, these data suggest that TCF7L2 expression during CNS myelination is restricted to a defined stage of oligodendroglial differentiation similar to the findings in murine CNS.

### 3. TCF7L2 expression in human demyelinating diseases and other inflammatory non-demyelinating diseases

**Figure 4:**
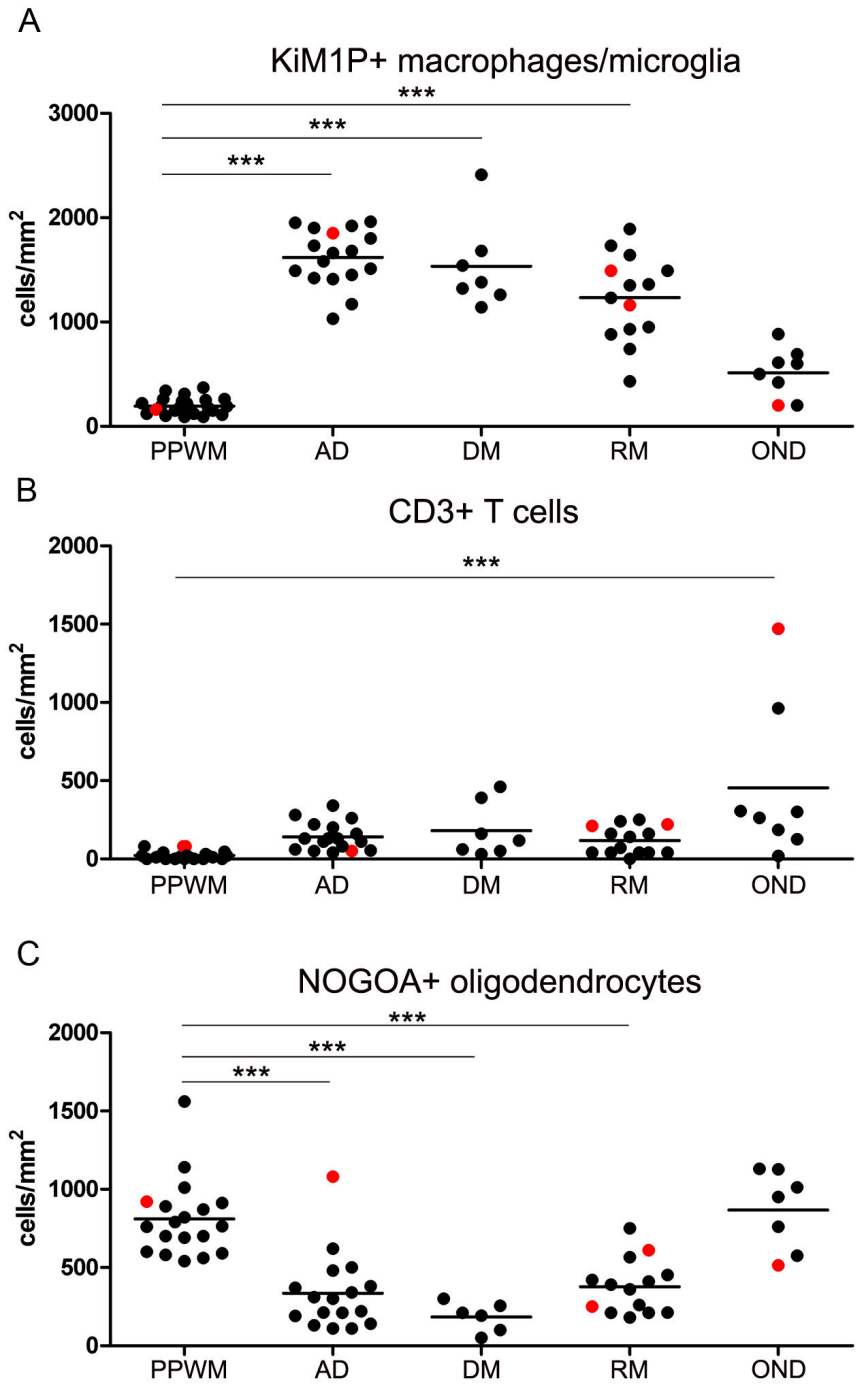
Quantification of macrophages/microglia, T cells, and oligodendrocytes in inflammatory demyelinating and non-demyelinating human CNS diseases. Tissue samples from patients with early MS and non-demyelinating inflammatory diseases were stained for macrophages/microglia (anti-KiM1P), T cells (anti-CD3) and oligodendrocytes (anti-NOGOA). Numbers of macrophages/microglia were significantly increased in all lesion areas compared to PPWM (**A**). T cells were highest in patients with inflammatory, non-demyelinating CNS diseases (**B**). Oligodendrocytes were significantly decreased in all MS lesion areas (**C**). The red dots (**A to C**) indicate tissue samples with high numbers of TCF7L2 expressing cells (see Figure 5); however no obvious difference between these tissue samples and the others were observed with respect to macrophages, T cells and oligodendrocytes. PPWM = periplaque white matter, AD = actively demyelinating, DM = demyelinated, RM = remyelinating/remyelinated, OND = other inflammatory neurological diseases.

**Figure 5:**
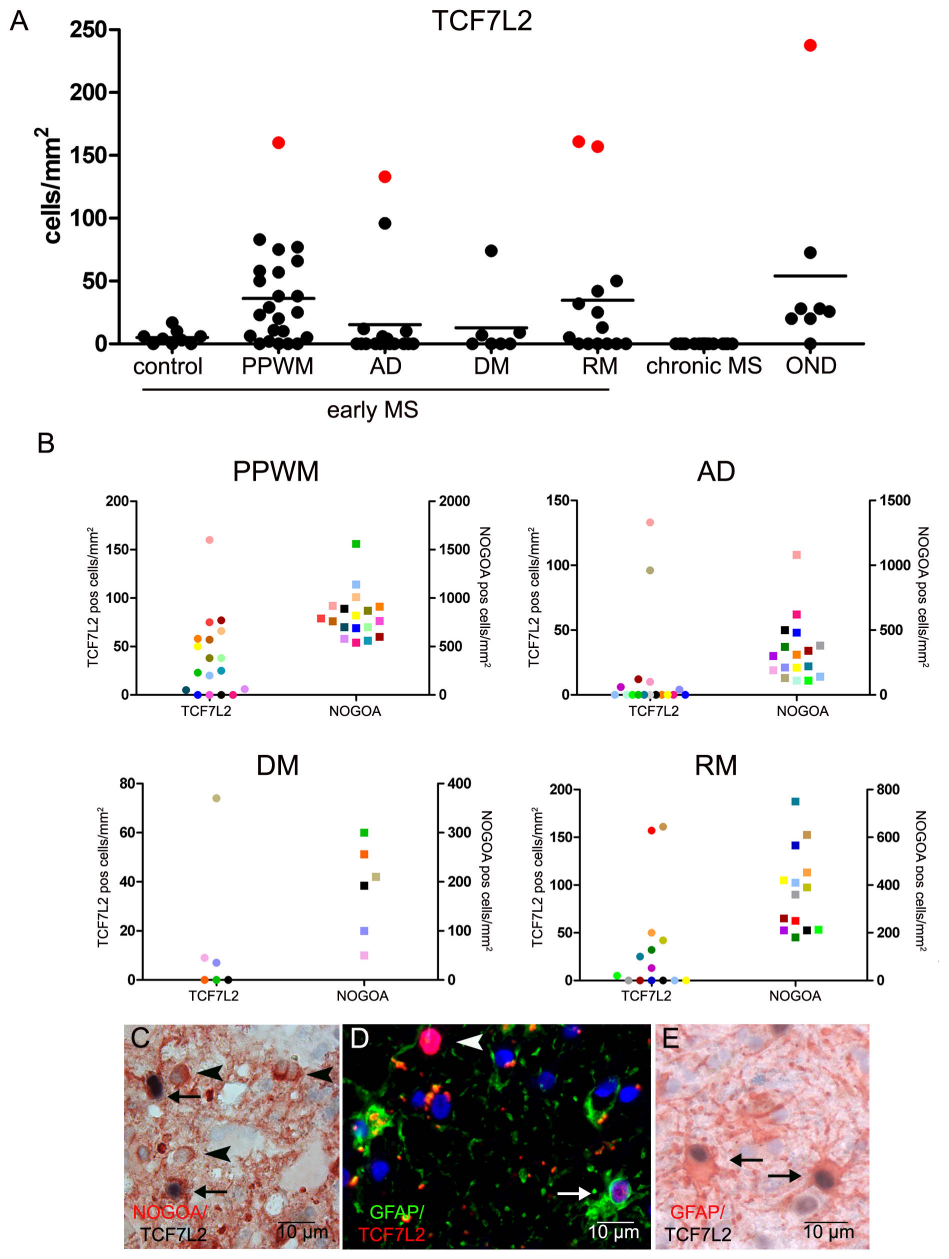
Expression of TCF7L2 in MS lesions and non-demyelinating inflammatory CNS diseases. In a subset of early MS lesions increased numbers of TCF7L2 positive cells were detected in the periplaque white matter and in remyelinating lesion areas. However, also in other inflammatory neurological diseases (OND) TCF7L2 positive cells were present. Red dots indicate a subset of tissue samples with high numbers of TCF7L2 expressing cells (see Figure 4) (**A**). There was no correlation between numbers of TCF7L2 and NOGOA positive cells in the different MS lesion areas. NOGOA positive oligodendrocytes and TCF7L2 expressing cells were quantified in periplaque white matter (PPWM), actively demyelinating (AD), demyelinated (DM) and remyelinating (RM) lesion areas; TCF7L2 expressing cells (dots) and NOGOA expressing cells (squares) from the same lesion area are labelled in the same colour (**B**). Double stainings revealed that a subset of TCF7L2 positive cells were NOGOA positive oligodendrocytes (arrows). TCF7L2 negative oligodendrocytes are indicated by arrow heads (Double immunohistochemistry for NOGOA (red) and TCF7L2 (black) (**C**). In inflammatory non demyelinating disease TCF7L2 positive oligodendrocytes and astrocytes were detected (**D** and **E**). In **D** a GFAP and TCF7L2 positive astrocyte (arrow) as well as a GFAP negative TCF7L2 positive cell (arrow head) are depicted (double immunohistochemistry for GFAP (green) and TCF7L2 (red). Additional GFAP and TCF7L2 positive cells are shown in **E** (arrows) (double immunohistochemistry for GFAP (red) and TCF7L2 (black)).

To further study the role of TCF7L2 expression in demyelinating and inflammatory CNS diseases we analyzed the expression of TCF7L2 in early and chronic MS lesions as well as in inflammatory non demyelinating tissue samples. Early MS lesions were characterized by a homogeneous infiltration of macrophages/microglia (between 1602 +/- 65 cells/mm^2^ in actively demyelinating and 1234/mm^2^ in remyelinating lesion areas); T cells were present in lower numbers (between 118 +/- 24 cells/mm^2^ in remyelinating and 181 +/- 66 cells/mm^2^ in demyelinated lesion areas) and were located perivascularly or within the parenchyma (Figure 4 A and B). Macrophages/microglia numbers were significantly lower in PPWM than lesions; however no significant differences were observed between actively demyelinating, demyelinated or remyelinated lesion areas in early MS lesions (Figure 4A). The numbers of oligodendrocytes were significantly reduced in all lesion areas compared to PPWM (Figure 4C). We detected TCF7L2-positive cells in 23 out of 33 tissue samples from patients with early MS. In early MS lesions the majority of TCF7L2-positive cells were found in the PPWM (36 +/- 8 cells/mm^2^) and remyelinating lesion areas (34 +/- 15 cells/mm^2^) whereas fewer cells were counted in actively demyelinating and demyelinated lesions areas (2.4 +/- 1.2 and 3.9 +/- 2.1 cells/mm^2^) (Figure 5A). In contrast, in control tissue samples from patients without neurological diseases, only single positive cells were found (mean 5 cells/mm^2^; range 0-16.7/mm^2^, n =9) as mentioned above. Double stainings with TCF7L2 and NOGOA, revealed that many TCF7L2 positive cells also expressed NOGOA (mean 34. +/- 11%, range 0-67%) (Figure 5C). However, also single TCF7L2- and GFAP positive cells were detected. When the lesions were stratified with respect to the presence or absence of remyelination within the biopsy specimen no significant differences were observed. Furthermore, no correlation between the numbers of TCF7L2- and NOGOA positive cells was found (Figure 5B). In tissue samples from four MS patients (patient no. 2, 6, 26 and 30, see Table 1) high numbers of TCF7L2 positive cells were detected (PPWM (n=1), actively demyelinating (n = 1) or remyelinating (n = 2) lesion areas) (Figure 5A); however no obvious differences with respect to numbers of T cells, macrophages or NOGOA positive oligodendrocytes were found (Figure 4A to C).

In chronic MS lesions (18 lesions from 12 patients) in which inflammation was limited to the lesion border (chronic active) or was almost completely absent (chronic inactive) no TCF7L2 expression was found (Figure 5A).

To determine whether the reexpression of TCF7L2 is restricted to demyelinating diseases such as MS, we stained ten tissue samples from eight patients with inflammatory but non demyelinating diseases (e.g. encephalitis, vasculitis). Macrophages/microglia numbers were significantly lower whereas T cell numbers were significantly higher in tissue samples from patients with non-demyelinating neurological diseases compared to patients with early MS lesions (Figure 4A and B). As expected, oligodendrocytes were not reduced in this set of tissue samples (Figure 4C). TCF7L2 positive cells were found in comparable numbers as in the periplaque white matter of early MS lesions (Figure 5A). Many TCF7L2 positive cells expressed NOGOA (mean 25. +/-12%, range 0-100%); additionally many TCF7L2 and GFAP double positive cells were detected (mean 34 +/-14%, range 0-100%) demonstrating that the expression of TCF7L2 is not restricted to the oligodendroglial lineage in inflammatory conditions (Figure 4C and D). In one tissue sample (patient no 53, Table 1) high numbers of TCF7L2-positive cells were observed, again no obvious difference in the extent or composition of the inflammatory infiltrate was observed (Figure 4A and B).

In summary, these data demonstrate that TCF7L2 is expressed in early but not chronic MS lesions. However, the TCF7L2 expression is not limited to demyelinating conditions or the oligodendroglial lineage.

### 4. Expression of HDAC2 in early MS lesions and healthy controls

**Figure 6:**
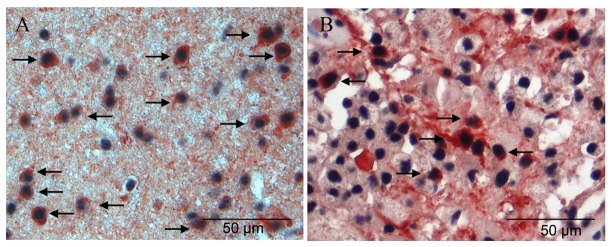
Expression of HDAC2 in MS lesions and control tissue samples. In control tissue samples (**A**) as well as in MS lesions (**B**) numerous NOGOA positive oligodendrocytes were seen which express abundantly HDAC2 (arrows) (double immunohistochemistry for NOGOA (red) and HDAC2 (black)).

Recent publications suggest that binding of TCF7L2 to β-catenin or HDAC1/2 either inhibits or promotes the differentiation of oligodendroglial lineage cells [19]. The presence and upregulation of β-catenin or other components of the wnt/β-catenin pathway in MS lesions have been described in earlier publications [29,30]. Therefore, we analyzed the expression of HDAC2 in a subset of MS lesions and in tissue samples from healthy controls. In control samples as well as in MS lesions numerous HDAC2-positive cells were observed by immunohistochemistry (Figure 6A and B). In MS tissue samples between 78 and 100% (mean 96 +/- 6%) of NOGOA expressing oligodendrocytes expressed also HDAC2 comparable to tissue samples from healthy controls (mean 94 +/- 6; range 83-100%).

In summary, these data indicate that HDAC2 is abundantly expressed in the nucleus of oligodendrocytes in early MS lesions.

## Discussion

Here, we demonstrate that the transcription factor TCF7L2 is expressed during development and downregulated in adulthood in oligodendroglial lineage cells. Furthermore, in demyelinating and inflammatory diseases TCF7L2 is reexpressed, but the expression of TCF7L2 is not restricted to oligodendrocytes. Furthermore, the potential TCF7L2 binding partner HDAC2 is abundantly expressed in oligodendroglial lineage cells.

TCF7L2 belongs to a subfamily of high mobility group (HMG)-box-containing superfamily of transcription factors. TCF7L2 binds to the Wnt response element; however for regulating transcription TCF7L2 requires additional cofactors (for review, see 31). Without β-catenin TCF7L2 acts as a transcriptional repressor, e.g. by binding Groucho/TLE (reviewed by [32]). Deficiency of TCF7L2 is associated with impaired oligodendroglial differentiation [19]. Misexpression of a dominant repressive TCF7L2 mutant that lacks the β-catenin binding site promotes ectopic expression of early oligodendroglial markers suggesting that a TCF7L2 mediated, β-catenin independent repressor interaction drives oligodendrocyte precursor formation [19]. During developmental myelination, we observed a temporally regulated expression of TCF7L2 in human and murine white matter which was limited to oligodendroglial lineage cells expressing OLIG2. This observation is in line with an earlier publication [18]. During myelination, many TCF7L2 positive cells express also NOGOA, a marker of mature oligodendrocytes that does not colocalizes with NG2 [28]. The presence of TCF7L2 positive cells that do not express NOGOA suggests that TCF7L2 is expressed in oligodendroglial lineage cells at the transition from immature to mature oligodendrocytes. This hypothesis is corroborated by the finding that TCF7L2 expression in the human and murine CNS peaks approximately at the same time point as myelination. During cuprizone induced de- and remyelination maximal TCF7L2 expression was observed at day 42 after onset of cuprizone feeding, a time point at which remyelination is initiated. In contrast, the recruitment of progenitors starts already during the cuprizone diet as indicated by the increase of OLIG2 or NOGOA positive oligodendroglial cells during late stages of cuprizone induced demyelination and as described by Matsushima and colleagues [27]. These findings suggest that TCF7L2 is upregulated during a defined differentiation stage of oligodendroglial lineage cells at the transition from OPCs to mature oligodendrocytes.

In human demyelinating diseases the situation is more complex. Earlier studies reported the expression of TCF7L2 in inflammatory MS lesions [18]. This finding together with the developmental observations suggested that expression of TCF7L2 might be used to identify differentiating or remyelinating oligodendrocytes; this hypothesis was supported by our finding that relatively high numbers of TCF7L2-positive cells were observed in a subset of remyelinating but not in actively demyelinating, demyelinated or chronic MS lesions. However, we observed TCF7L2 positive cells only in 8 out of 15 lesion areas with remyelination; this might be explained by the fact that expression of TCF7L2 is restricted to a narrow time window during oligodendroglial differentiation. The presence of TCF7L2 positive oligodendrocytes in tissue samples from patients with an inflammatory but not demyelinating disease may suggests that also in these inflammatory conditions OPCs differentiate into oligodendrocytes due to an inflammation induced oligodendroglial turnover as it has been reported for experimental models of injury [33] (for review, see 34). Surprisingly, we found also expression of TCF7L2 in astrocytes in human demyelinating and inflammatory disease that appeared weaker than in oligodendrocytes. The biological significance of this finding is unclear; since so far little is known about the functional role of TCF7L2 in astrocytes [35].

TCF7L2 is a well-known co-activator of β-catenin and activation of the canonical wnt/β-catenin pathway in vitro or in vivo impairs oligodendroglial differentiation [18,19,36–38]. The presence of members of the wnt/β-catenin pathway in MS lesions as well as recent observations that enforced constitutive expression of β-catenin inhibited myelination and differentiation of oligodendrocytes led to the suggestion that a disturbed wnt/β-catenin-pathway may contribute to remyelination failure in chronic MS lesions [18,19,30,39]. Furthermore, in animal studies prevention of the translocation of β-catenin to the nucleus promotes remyelination and myelination [29]. Without β-catenin TCF/LEFs assemble alternative complexes with other transcriptional co-repressors, such as Groucho/TLE or Smad3 [32,40]. Recent findings suggest that HDAC1 and 2 may compete with β-catenin for the binding to TCF7L2 thereby antagonizing the effect of wnt signaling and promoting oligodendroglial differentiation [19]. In our tissue samples we found a strong expression of HDAC2 in oligodendrocytes during CNS myelination, in healthy controls and in MS lesions. Therefore, it cannot be excluded that TCF7L2 binds to HDACs in oligodendrocytes resulting in the differentiation of oligodendrocytes, expression of myelin genes and initiation of remyelination at least in a subset of MS lesions.

In summary, we have shown that TCF7L2 is expressed during development and early MS lesion stages in oligodendrocytes. However, the expression of TCF7L2 is neither limited to oligodendrocytes nor to demyelinating conditions. Further studies are required to dissect further the functional role of TCF7L2 in oligodendrocytes and astrocytes during remyelination and myelination.
